# The combination of *Butyricicoccus pullicaecorum* and 3-hydroxyanthranilic acid prevents postmenopausal osteoporosis by modulating gut microbiota and Th17/Treg

**DOI:** 10.1007/s00394-024-03400-3

**Published:** 2024-05-16

**Authors:** Fuping Zhu, Hui Liu, Yinsheng Cao, Bing Dai, Hang Wu, Wuping Li

**Affiliations:** 1 Department of Foot and Ankle Orthopedics, The First Hospital of Hunan University of Chinese Medicine, Changsha, 410007 Hunan China; 2 Department of Orthopedic Surgery, The Second Xiangya Hospital of Central South University, Changsha, 410011 Hunan China; 3 Department of Pharmacy, The First Hospital of Hunan University of Chinese Medicine, Changsha, 410007 Hunan China

**Keywords:** Postmenopausal osteoporosis, *Butyricicoccus pullicaecorum*, 3-Hydroxyanthranilic acid, Gut microbiota, Th17, Treg

## Abstract

**Background:**

Postmenopausal osteoporosis (PMO) is a chronic condition characterized by decreased bone strength. This study aims to investigate the effects and mechanisms of the combination of *Butyricicoccus pullicaecorum* (*Bp*) and 3-hydroxyanthranilic acid (3-HAA) on PMO.

**Methods:**

The effects of *Bp* and 3-HAA on PMO were evaluated in ovariectomized (OVX) rats by assessing stereological parameters, femur microstructure, and autophagy levels. The T helper (Th) 17/Regulatory T (Treg) cells of rats were detected using flow cytometric analysis. Furthermore, the impact of *Bp* and 3-HAA on the gut microbiota of rats was assessed using 16S rRNA gene sequencing. The correlation between the gut microbiota of rats and Th17/Treg immune factors, as well as femoral stereo parameters, was separately assessed using Spearman rank correlation analysis.

**Results:**

*Bp* and 3-HAA treatments protected OVX rats by promoting osteogenesis and inhibiting autophagy. Compared to the Sham group, OVX rats showed an increase in Th17 cells and a decrease in Treg cells. *Bp* and 3-HAA reversed these changes. *Enterorhabdus* and *Pseudomonas* were significantly enriched in OVX rats. *Bp* and 3-HAA regulated the gut microbiota of OVX rats, enriching pathways related to nutrient metabolism and immune function. There was a correlation between the gut microbiota and the Th17/Treg, as well as femoral stereo parameters. The concurrent administration of Bp and 3-HAA medication facilitated the enrichment of gut microbiota associated with the improvement of PMO.

**Conclusion:**

The combination therapy of *Bp* and 3-HAA can prevent PMO by modulating the gut microbiota and restoring Th17/Treg immune homeostasis.

## Introduction

Postmenopausal osteoporosis (PMO) is a systemic chronic skeletal disorder that occurs in postmenopausal women [1]. The characteristic manifestations of PMO include decreased bone mineral density and deterioration of bone structure, which ultimately lead to decreased bone strength and increased susceptibility to fractures [2]. Due to the potential consequences of fractures, such as decreased quality of life, disability, and increased economic burden, the prevention and treatment of PMO have gained significant attention in clinical practice [3, 4]. Postmenopausal bone loss is primarily associated with the discontinuation of estrogen during menopause. Other known high-risk factors include advanced age, smoking, low body weight, and genetic predisposition [5]. Currently, the primary management strategies for PMO include non-pharmacological therapies and pharmacological interventions [6]. Non-pharmacological strategies for PMO management include fall prevention and lifestyle modifications. Pharmacological treatment options for PMO primarily include vitamin D and calcium supplementation, bisphosphonates, synthetic parathyroid hormone, and monoclonal antibodies such as Romosozumab [7, 8]. Nevertheless, the current pharmacological treatments for PMO are associated with various adverse reactions, and improvements are still needed in terms of prognosis [9]. In recent years, using probiotics as an adjuvant therapy or in combination with medications has gradually gained recognition among individuals [10].

T-helper 17 (Th17) and regulatory T (Treg) cells are two distinct types of T cells that can interact with each other and regulate the differentiation and formation of osteoclasts [11]. In vivo and in vitro, Treg cells inhibit the differentiation of osteoclasts, while Th17 cells promote osteoclast differentiation. In PMO mice, the percentage of the Th17 subset and the Th17/Treg ratio are increased, while the percentage of the Treg subset is significantly reduced [12]. Interleukin (IL)-17 is produced by Th17 cells, and its interaction with IL-10, released by Treg cells, can collectively modulate immune responses [13]. According to reports, anti-IL-17 treatment can prevent estrogen deficiency-induced bone destruction [14]. On the other hand, the induction of osteoclast formation requires macrophage colony-stimulating factor (M-CSF) and receptor activator of nuclear factor-κB ligand (RANKL). Th17 cells release RANKL in a soluble form, while Treg cells inhibit the levels of RANKL and M-CSF [15]. 1,25-Dihydroxy vitamin D3 may exert a bone-protective effect in patients with corticosteroid-treated rheumatoid arthritis by regulating Th17 polarization, inhibiting Th17 cell cytokines, and stimulating IL-4 [16]. Therefore, modulating the Th17/Treg immune balance associated with PMO may contribute to the improvement of bone loss.

Probiotics are dietary supplements composed of beneficial microorganisms. When given in adequate amounts, they can confer health benefits to the host [17]. Recent studies have suggested that probiotics can be beneficial to host bone metabolism through the modulation of gut microbiota [18]. According to the pathogenesis of PMO, vitamin D analogs (such as calcitriol) and calcium supplements are widely used in clinical treatment for PMO. Combining Probio-M8 with conventional treatment has significantly improved bone metabolism in PMO patients. Its beneficial effects may be attributed to the modulation of gut microbiota and its associated metabolic pathways [10]. *Butyricicoccus pullicaecorum* (*Bp*) is a gut bacterium that produces butyrate, and it can regulate the expression of short-chain fatty acids (SCFAs) transporters and receptors [19]. The *Bp* 25-3 strain does not disrupt the composition and metabolic activity of the gut microbiota and demonstrates good safety and tolerance in healthy individuals [20]. *Lactobacillus rhamnosus GG* (*LGG*) exerts a therapeutic effect on PMO through improvements in Th17/Treg balance and alleviation of inflammation and mucosal damage induced by estrogen deficiency [21]. *Bp* and SCFAs are involved in the restoration of intestinal Th17/Treg homeostasis and the regulation of inflammation-associated damage [22]. There is currently no specific research on the use of *Bp* strains in treating PMO.

3-Hydroxyanthranilic acid (3-HAA) is a metabolite of tryptophan that has been shown to possess immunomodulatory properties and regulate lipid metabolism [23]. The research findings suggest that a decrease in the concentration of 3-HAA may be one of the reasons for bone loss [24]. In patients with symptomatic hand osteoarthritis, the level of 3-HAA in the plasma is decreased [25]. In vitro, the depletion of Th17 cells can be directly mediated by 3-HAA, a metabolite of indoleamine 2,3-dioxygenase (IDO) generated through the proximal degradation of tryptophan. [26]. 3-HAA is a biomarker associated with the renal protective effects of *Tripterygium wilfordii* in rheumatoid arthritis rats [27], and is associated with gut microbiota metabolism [28]. However, the therapeutic effect of 3-HAA on PMO remains to be investigated.

Based on this, it is hypothesized that *Bp* and 3-HAA may have a combined influence on gut microbiota and Th17/Treg immune homeostasis in PMO. However, the specific mechanisms of action still require further confirmation. The purpose of this study is to examine the effects of the combination of the probiotic strain *Bp* and 3-HAA in PMO rats. The aim is to provide novel therapeutic strategies for the improvement of osteoporosis. Our study is expected to provide new strategies for clinical PMO probiotic combination therapy.

## Methods and materials

### Animal and treatments

Fifty adult female Sprague–Dawley (SD) rats weighing 280–320 g were obtained from Hunan SJA Laboratory Animal Co., Changsha, China. SD rats were housed under specific pathogen-free conditions. The rats were housed in a standard laboratory environment with a temperature of 25 ± 2 °C, humidity of 50 ± 5%, and a 12-h light/dark cycle. The SD rats were randomly divided into 5 groups, with 10 in each group. Five rats were randomly assigned to each metabolic cage, with any two cages forming a group. Animals were provided with a standard chow diet with free access to water. The rats were anesthetized with an intraperitoneal injection of xylazine (5 mg/kg) and ketamine (60 mg/kg). In the ovariectomized (OVX) group, the rats underwent bilateral ovariectomy surgery. In the sham group, the rats were sham-operated. In the OVX+3-HAA group, OVX rats received treatment with 3-HAA (120 mg/kg, administered via tail vein injection once weekly) for a duration of 12 weeks [29]. In the OVX+*Bp* group, OVX rats received treatment with *Bp* (Bio-129459, Biobw, China) for 12 weeks. According to the reference [30], the *Bp* 25-3 T (LMG 24109 T; CCUG 55265 T, Bio-129459, Biobw) concentration used was 1.09 × 10^9^ colony-forming units (CFU)/mL, which corresponds to a dosage of 4.55 × 10^9^ CFU/kg. *Bp* was administered orally via gavage. The OVX+3-HAA+*Bp* group received a combination treatment of 3-HAA and *Bp* for a duration of 12 weeks. After 12 weeks of treatment, blood samples were collected. Subsequently, the rats were euthanized by intraperitoneal injection of 150 mg/kg pentobarbital, and spleen tissues were harvested for immune cell analysis. After the femur was removed from the rats, cartilage, adhering tissues, and bone marrow were cleared. Finally, the left femur of each rat was stored at −80 °C for gene expression analysis. The right femur was preserved in 4% paraformaldehyde (BL539A, Biosharp, China) for histological measurements. All experiments were approved by the ethics committee.

### The femur stereological parameters assay

According to the previous study [31], the primary volume (V primary) of the whole femur was measured using the immersion method. The femurs were decalcified and processed for histology. Based on a reference study [32], we obtained randomly oriented femoral sections using the Orientator method. Eight sections were obtained from each femoral head. We used a trephine needle to punch out a non-spherical section from the femoral plate and estimated its area and diameter. We prepared 5 µm and 20 µm sections from all test plates of rat femurs, which were embedded in the same paraffin block as the circular sections. According to the following formula, we stained the plates with hematoxylin and eosin (H&E) and performed a subsequent area analysis of the circular fragments to determine bone shrinkage. Volume shrinkage (Vsh) = 1 − (after area/before area)^1.5^. The final total bone volume can be calculated according to the following formula. V (final) = (1 − Vsh) × V (primary). According to the previous study [33], the volume of trabeculae was calculated using the Delesse formula and the point counting method. Based on the Disector method and relevant formulas [34], the numerical density (number of cells per unit volume of trabeculae) was determined on 20 µm thick sections. According to a previous study [35], we used to scan optical planes and unbiased counting frames to perform a secondary sampling on each section. The target sample contains bone cells. If the cell nucleus is completely or partially within the counting frame, the cells should be sampled and counted based on the inclusion rule (touching the upper or right line) or the exclusion rule (not touching the lower or left boundary).

### H&E staining

The femurs were fixed in 4% paraformaldehyde and decalcified using 10% ethylene diamine tetra acetic acid (EDTA, Genview Biotech, China). Following decalcification, the femurs were embedded in paraffin. The femurs were sectioned with a thickness of 4 μm. According to the previous study [36], the sections were stained with H&E. The bone tissue microstructure was observed under an optical microscope (BA410T, Motic, China).

### Enzyme-linked immunosorbent assay (ELISA)

From the manufacturer’s instructions, the concentrations of estradiol (E2), bone Gla-protein (BGP), IL-10, and IL-17 in the peripheral blood of the rats were measured using detection kits. The ELISA kits used included E2 (CSB-E05110r, CUSABIO, China), BGP (CSB-E05129r, CUSABIO, China), IL-10 (KE20003, Proteintech, USA), and IL-17 (CSB-E07451r, CUSABIO, China).

### Western blot (WB) analysis

RIPA lysate (AWB0136, Abiowell, China) was used to extract proteins from the tissues. Protein concentration was determined using the bicinchoninic acid (BCA) protein assay kit (23227, Thermo Fisher, USA). The proteins were separated using SDS-PAGE gel (10%) and then transferred to a polyvinylidene fluoride membrane. After blocking with skimmed milk for 90 min, the primary antibody was used to incubate the membrane overnight. The primary antibodies used included anti-LC3B (1:3000, 14600-1-AP, Proteintech, USA), anti-Caspase-3 (1:1000, 19677-1-AP, Proteintech, USA), anti-Bcl-2 (1:5000, 60178-1-Ig, Proteintech, USA), anti-p-mTOR (1:5000, ab109268, Abcam, UK), anti-mTOR (1:10,000, ab134903, Abcam, UK), and anti-β-actin (1:5000, 66009-1-Ig, Proteintech, USA). The anti-β-actin antibody was used to normalize the results. HRP-conjugated goat anti-mouse IgG (1:5000, SA00001-1, Proteintech, USA) and HRP-conjugated goat anti-rabbit IgG (1:6000, SA00001-2, Proteintech, USA) were applied to incubate the membrane for 90 min. The chemiluminescence method (Millipore, USA) was used for visualization. The imaging analysis was conducted using Quantity One software (Quantity One v4.6.6, Biorad, America).

### Cell isolation

A single-cell suspension of rat spleen tissue was prepared using a 40 μm wetted cell strainer. After centrifugation, the pellet containing red blood cells was lysed using a red blood cell lysis buffer (AWC0390a, Abiowell, China). The suspension was allowed to settle for 10 min. Mononuclear cells were collected by centrifugation. A mononuclear cell suspension from rat peripheral blood was prepared using a red blood cell lysis buffer and rat peripheral blood mononuclear cell (PBMC) isolate kit (P9160, Solarbio, China). The mononuclear cells obtained after centrifugation were used for flow cytometry analysis.

### Flow cytometry analysis

To identify Th17 cells, we first stimulated the PBMCs with a Cell Stimulation Cocktail (containing phorbol 12-myristate 13-acetate, ionomycin) in the presence of protein transport inhibitors. The cells were then treated with a cell permeabilization reagent (420801, Biolegend, USA). Subsequently, the lymphocytes were stained with anti-CD4 FITC (11-0040-82, eBioscience, USA) and anti-IL-17 APC (17-7177-81, eBioscience, USA) antibodies for cell labeling.

To identify Treg cells, we treated the cells with a cell permeabilization reagent (420801, Biolegend, USA). After that, the lymphocytes were stained with anti-CD4 FITC, anti-CD25 APC (17-0390-82, eBioscience, USA), and anti-Foxp3 PE (12-5773-82, eBioscience, USA) antibodies for cell labeling. The percentages of CD4+CD25+Foxp3+ (Treg) cells and CD4+IL-17+ (Th17) cells were detected using a flow cytometer (A00-1-1102, Beckman, USA).

### Reverse transcription-quantitative polymerase chain reaction (RT-qPCR)

According to the manufacturer’s instructions, total RNA was extracted using Trizol (15596026, Thermo Fisher Scientific, USA). The cDNA sequences were generated using the mRNA reverse transcription kit (CW2569, CWBIO, China). RNA relative quantification analysis was performed using the UltraSYBR Mixture (CW2601, CWBIO, China). The amplification conditions were set: 95 °C for 10 min for initial denaturation, followed by 40 amplification cycles at 95 °C for 15 s and 60 °C for 35 s. The mRNA expression was calculated using the 2^−ΔΔCt^ method. β-actin was the normalization. The primer sequences used were sourced from the NCBI database and are shown in Table 1.

**Table 1:** Primer information for RT-qPCR

Gene	Primer sequence
β-actin	Forward primer: 5′ACATCCGTAAAGACCTCTATGCC3′
	Reverse primer: 3′TACTCCTGCTTGCTGATCCAC5′
Foxp3	Forward primer: 5′TTCGCCTACTTCAGAAACCACC3′
	Reverse primer: 3′CAAATTCATCTACGGTCCACACT5′
IL-10	Forward primer: 5′AATAAGCTCCAAGACAAAGGT3′
	Reverse primer: 3′TCACGTAGGCTTCTATGCAG5′
TGF-β	Forward primer: 5′ACTACGCCAAAGAAGTCACC3′
	Reverse primer: 3′CACTGCTTCCCGAATGTCT5′
IFN-γ	Forward primer: 5′CAACCAGGCCATCAGCAAC3′
	Reverse primer: 3′CCCAGAATCAGCACCGACT5′
IL-6	Forward primer: 5′TCACTATGAGGTCTACTCGG3′
	Reverse primer: 3′CATATTGCCAGTTCTTCGTA5′
IL-2	Forward primer: 5′CCAAGCAGGCCACAGAATTG3′
	Reverse primer: 3′TCCAGCGTCTTCCAAGTGAA5′
IL-17	Forward primer: 5′CGTTTCCTCTATTGTCCGCCAT3′
	Reverse primer: 3′TGGAAGGCAGACAATTCTAACCC5′

### Gut microbiota sequencing analysis

Fifty fresh fecal samples (5 groups, *n* = 10 rats/group) were collected in sterile tubes and stored in liquid nitrogen. Following the instructions of the manufacturer, DNA extraction from fecal samples was performed using the HiPure Stool DNA Kits (Magen, Guangzhou, China). Gel electrophoresis was then carried out to detect the bacterial genomic DNA. Based on the previous study [37], the gut bacterial composition of the 5 groups of samples was analyzed using 16S rDNA gene analysis. The DNA was subjected to 16S amplicon sequencing using the NovaSeq 6000 platform (Illumina, USA). The sequencing strategy used was PE250 paired-end sequencing, and the raw data was obtained after the sequencing run. The raw data was subjected to quality control, and valid data was obtained. We used the Quantitative Insights into Microbial Ecology 2 (Qiime2, USA) software to determine and analyze DNA sequences in the microbial community using the naive Bayes classifier and q2-feature classifier. We obtained Amplicon Sequence Variant (ASV) identifiers for each microorganism and statistically analyzed the community composition of each sample at various levels. The database used was silva-138-99. A Venn diagram was drawn. The differences between the gut microbiota composition among the groups were analyzed based on the Beta diversity indices. The figures were produced using R (V3.5.1, Rstudio, USA). We employed the phylogenetic investigation of communities by reconstruction of unobserved states (PICRUSt, V1.0.0, Huttenhower Lab, USA) software for targeted functional prediction analysis of the microbial community. By integrating the Kyoto Encyclopedia of Genes and Genomes (KEGG) pathway information with gene data, we could predict the pathway status of the entire community.

#### Statistical analysis

Statistical analysis was conducted using GraphPad Prism 8.0 software (GraphPad Software, USA). The data are presented as mean ± SD. Statistical differences were analyzed using one-way analysis of variance (ANOVA) followed by post-hoc Tukey’s test. The Beta diversity values of the samples were calculated using the Anosim non-parametric test. Spearman correlation analysis was performed to illustrate the relationships among the parameters. A *P* value less than 0.05 was considered statistically significant.

## Results

### The combined use of *Bp* and 3-HAA improved the stereological parameters of the femur in OVX rats

**Fig. 1:**
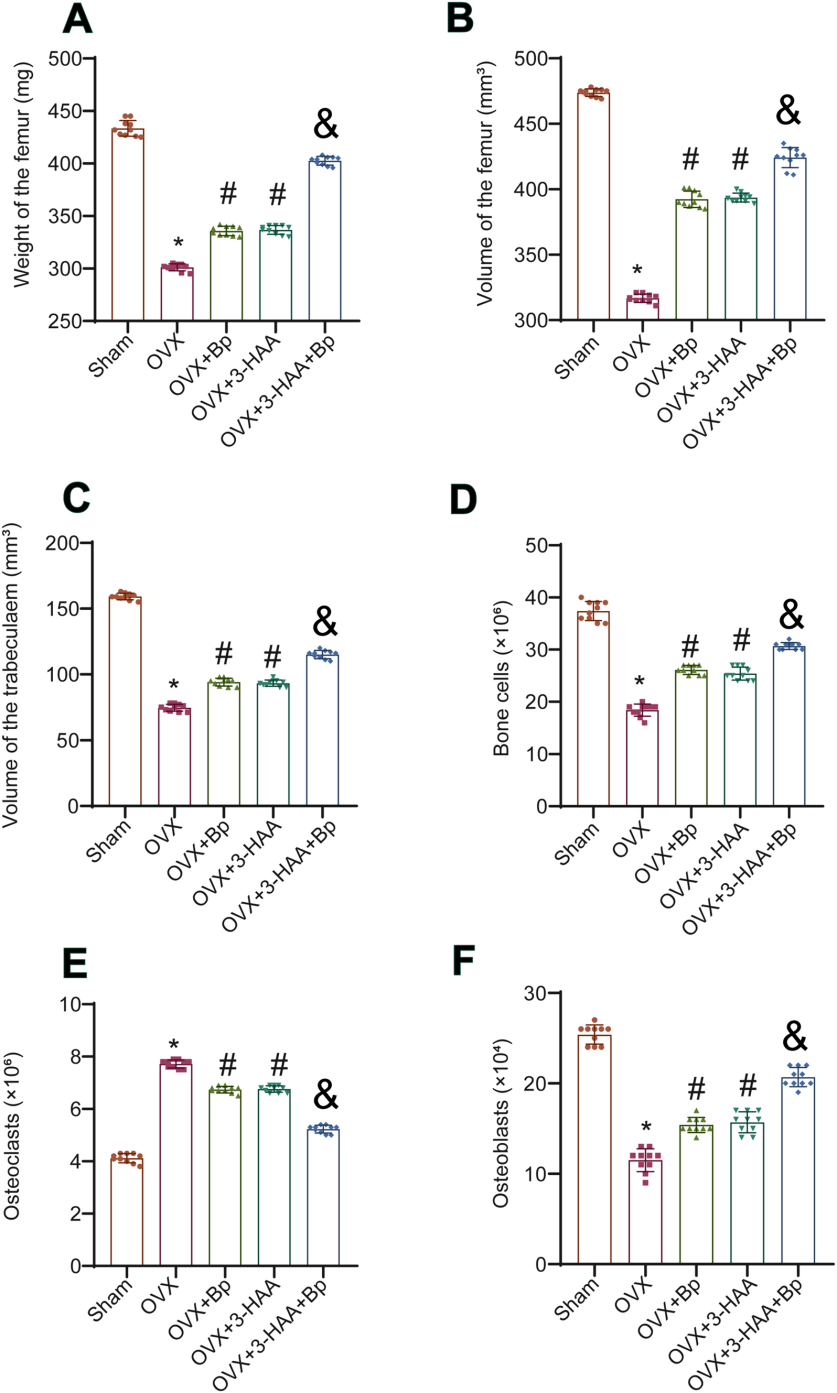
Improvement of femoral stereological parameters in OVX rats with the combined use of *Bp* and 3-HAA. **A** Femoral mass was measured in rats. **B** Femoral volume was measured in rats. **C** Femoral trabecular volume was assessed. **D** Total number of bone cells was measured. **E** Total number of osteoclasts was calculated. **F** Total number of osteoblasts was measured. *n* = 10, * *P* < 0.05 vs. Sham, ^#^ *P* < 0.05 vs. OVX, ^&^ *P* < 0.05 vs. OVX+*Bp*/OVX+3-HAA

To investigate the effect of the combined use of *Bp* and 3-HAA on femoral stereological parameters in PMO rats, we evaluated a series of femoral stereological parameters in OVX rats. Compared to the Sham group, the OVX group exhibited decreased femoral mass and volume. In contrast to the OVX group, administration of either *Bp* or 3-HAA alone increased femoral weight and volume in OVX rats. The femoral mass and volume were significantly increased in the OVX rats administered with a combination of *Bp* and 3-HAA compared to the groups receiving *Bp* or 3-HAA alone (Fig. 1A, B). Additionally, the trabecular volume of the femur was decreased in the OVX group compared to the Sham group. Administration of either *Bp* or 3-HAA alone in OVX rats increased the trabecular volume. Moreover, the combination treatment of *Bp* and 3-HAA resulted in a more significant increase in the trabecular volume compared to the individual treatments (Fig. 1C). The results of the bone cell count showed that the total number of bone cells and osteoblasts decreased, while the number of osteoclasts increased in the OVX group compared to the Sham group. In comparison to the OVX group, the OVX+*Bp* or OVX+3-HAA groups exhibited an increase in the total number of bone cells and osteoblasts, along with a decrease in osteoclasts. Moreover, the OVX+3-HAA+*Bp* group showed further increases in the total number of bone cells and osteoblasts, as well as a reduction in the number of osteoclasts compared to the OVX+*Bp* or OVX+3-HAA groups (Fig. 1D–F). From these observations, it is evident that both *Bp* and 3-HAA can improve the trabecular parameters of the femur in OVX rats. Furthermore, the combination of these two drugs yields a more pronounced effect.

### The combined use of *Bp* and 3-HAA improved femoral pathology and autophagy in OVX rats

**Fig. 2:**
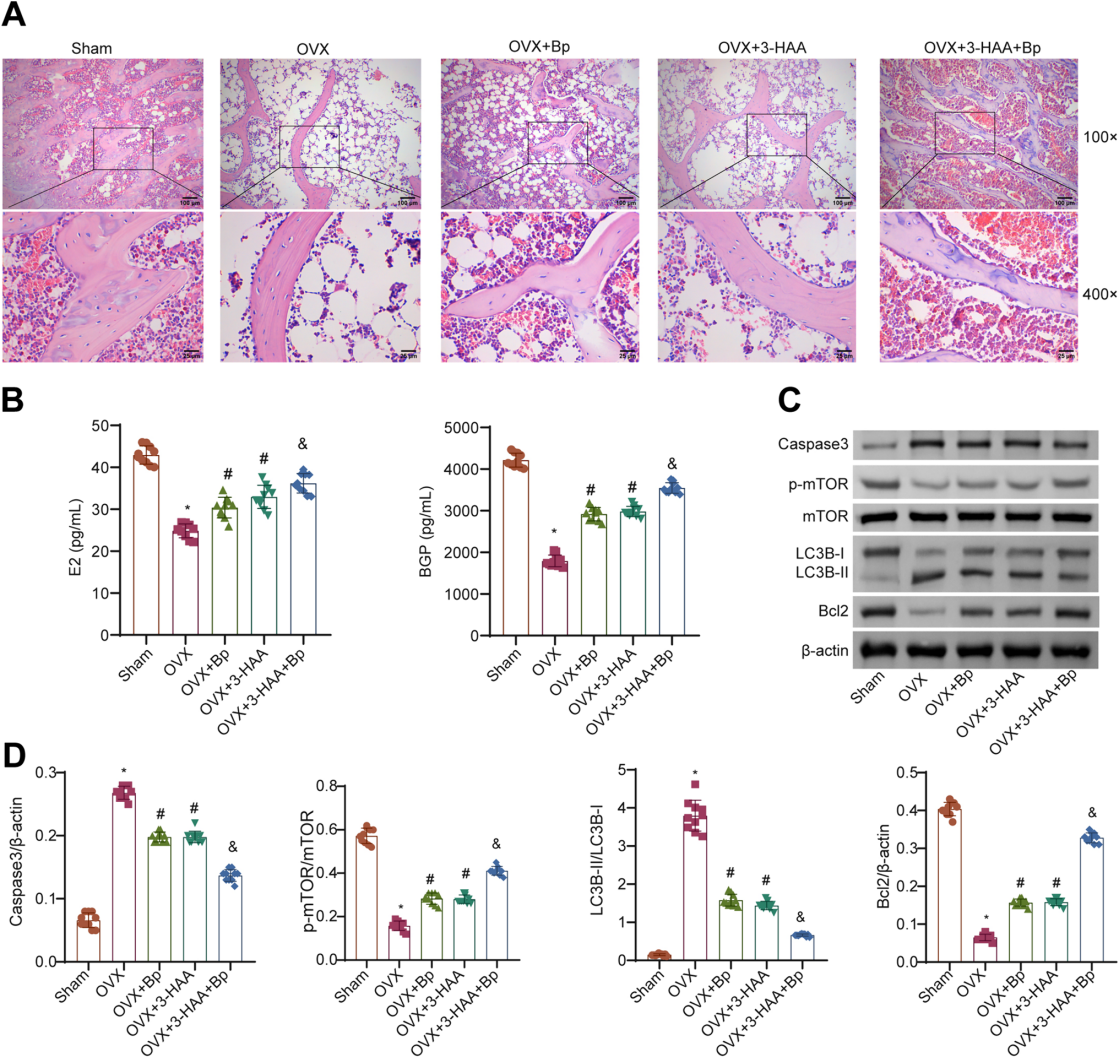
Combined use of *Bp* and 3-HAA alleviates femoral pathology and autophagy in OVX rats. **A** H&E staining of femoral tissue in rats. **B**. Measurement of serum levels of E2 and BGP. **C** WB analysis of protein bands of autophagy-related factors. **D** Evaluation of the expression levels of autophagy-related factors in rats. *n* = 10, * *P* < 0.05 vs. Sham, ^#^ *P* < 0.05 vs. OVX, ^&^ *P* < 0.05 vs. OVX+*Bp*/OVX+3-HAA

Then, we evaluated the effects of the combined use of *Bp* and 3-HAA on the pathological structures and autophagic levels in the femurs of OVX rats. The results showed that compared to the Sham group, the femoral trabeculae in the OVX group of rats were severely damaged, showing extensive areas of cavitation. In comparison to the OVX group, the severity of trabecular damage and the number of bone tissue cavities were reduced in the OVX+*Bp* or OVX+3-HAA groups. Furthermore, the OVX+3-HAA+*Bp* group exhibited even less pathological damage relative to the OVX+*Bp* or OVX+3-HAA groups (Fig. 2A). Compared to the Sham group, the levels of serum E2 and BGP were decreased in the OVX rats. After treatment with either *Bp* or 3-HAA, an elevation in serum levels of E2 and BGP was observed in OVX rats. Furthermore, in comparison to the administration of either drug alone, a higher increase in serum levels of E2 and BGP was noted in OVX rats that were co-administered with *Bp* and 3-HAA (Fig. 2B). Compared to the Sham group, the levels of autophagy markers LC3B-II/LC3B-I and Caspase3 were elevated in the femoral tissue of the OVX group rats, while the levels of autophagy-regulating molecules Bcl2 and p-mTOR/mTOR were downregulated. After administration of *Bp* or 3-HAA to OVX rats, the levels of LC3B-II/LC3B-I and Caspase3 were reduced, while the levels of Bcl2 and p-mTOR/mTOR were increased. The effect was more pronounced in OVX rats treated with the combined use of *Bp* and 3-HAA compared to the groups treated with *Bp* or 3-HAA alone (Fig. 2C, D). Therefore, the combined use of *Bp* and 3-HAA could alleviate femoral pathological damage and inhibit autophagy in OVX rats.

### The combined use of *Bp* and 3-HAA improved Th17/Treg immune balance in OVX rats

**Fig. 3:**
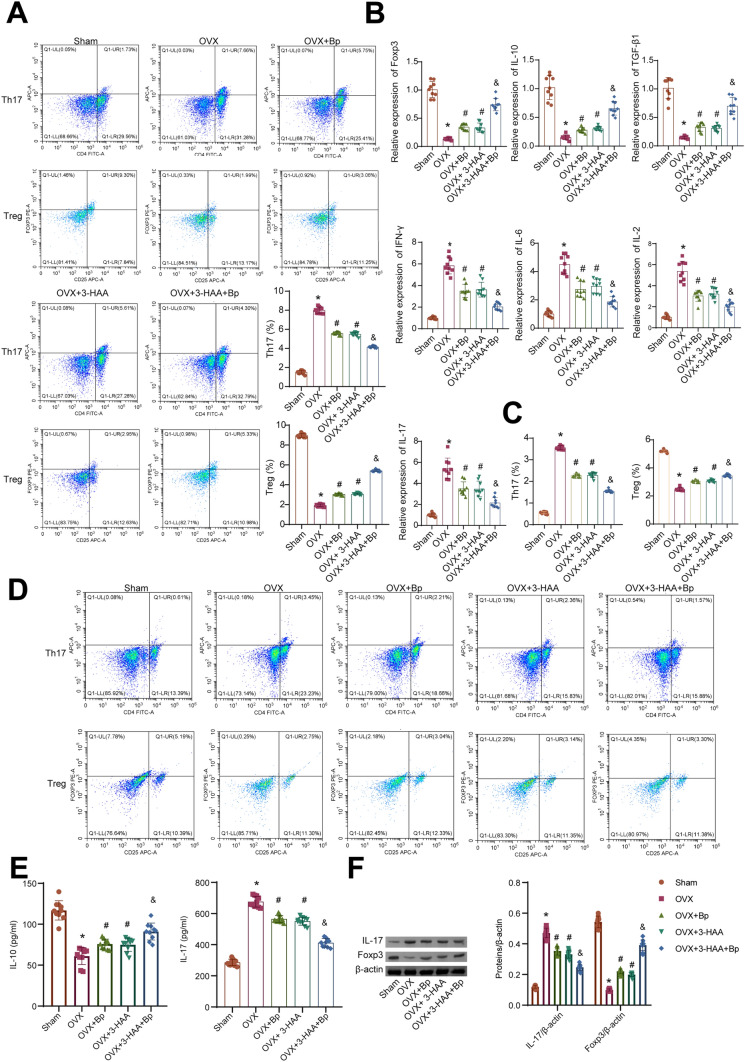
Combined administration of *Bp* and 3-HAA effectively improves the Th17/Treg balance in OVX rats. **A** The proportions of Th17 and Treg cells in spleen tissue were detected by flow cytometry. **B** The expression levels of IFN-γ, IL-6, IL-2, IL-17, Foxp3, IL-10, and TGF-β1 in spleen tissue were detected by RT-qPCR. C. Histograms showing the proportions of Th17 and Treg cells in peripheral blood. **D** The proportions of Th17 and Treg cells in peripheral blood were detected by flow cytometry. **E** The secretion levels of IL-17 and IL-10 in peripheral blood were detected by ELISA. F. Levels of IL-17 and Foxp3 in the femoral tissue were detected by WB. *n* = 10, * *P* < 0.05 vs. Sham, ^#^ *P* < 0.05 vs. OVX, ^&^ *P* < 0.05 vs. OVX+*Bp*/OVX+3-HAA

To assess whether the combined treatment of Bp and 3-HAA can improve Th17/Treg immunity in OVX rats, we evaluated the proportions of Th17/Treg cells and levels of related immune factors in each group. Both spleen tissue and peripheral blood of the rats were tested. Compared to the Sham group, an increase in the proportion of Th17 cells and a decrease in the proportion of Treg cells in spleen tissue was observed in OVX rats. However, after administration of *Bp* or 3-HAA to OVX rats, the opposite changes were observed. OVX rats treated with the combination of *Bp* and 3-HAA exhibited a consistent result with those treated with a single drug, but the effect was more pronounced (Fig. 3A). Compared to the Sham group, OVX rats exhibited increased expression levels of IFN-γ, IL-6, IL-2, and IL-17, while the levels of Foxp3, IL-10, and TGF-β1 were decreased in the spleen tissue. However, after administration of *Bp* or 3-HAA to OVX rats, the expression levels of IFN-γ, IL-6, IL-2, and IL-17 decreased, while the levels of Foxp3, IL-10, and TGF-β1 increased in the spleen tissue. In comparison to the groups treated with a single drug, the changes in the expression levels of Th17/Treg immune-related factors in the spleen tissue of OVX rats treated with the combined administration of *Bp* and 3-HAA were consistent. Still, the effect was more significant (Fig. 3B). The proportions of Th17/Treg cells and the levels of cytokines (IL-10, IL-17) in the peripheral blood of the 5 groups of rats corresponded to the results observed in the spleen tissue (Fig. 3C–E). Compared to the Sham group, OVX rats showed increased levels of IL-17 and decreased levels of Foxp3 in the femurs. Treatment with Bp or 3-HAA could reduce the levels of IL-17 in the femurs of OVX rats and increase the levels of Foxp3, with a more significant effect observed with combined treatment. Based on the findings mentioned above, it can be speculated that the combined administration of *Bp* and 3-HAA exerts inhibitory effects on the differentiation of Th17 cells while simultaneously promoting the differentiation of Treg cells.

### The combined use of *Bp* and 3-HAA improved the structure and functionality of gut microbiota in OVX rats

**Fig. 4:**
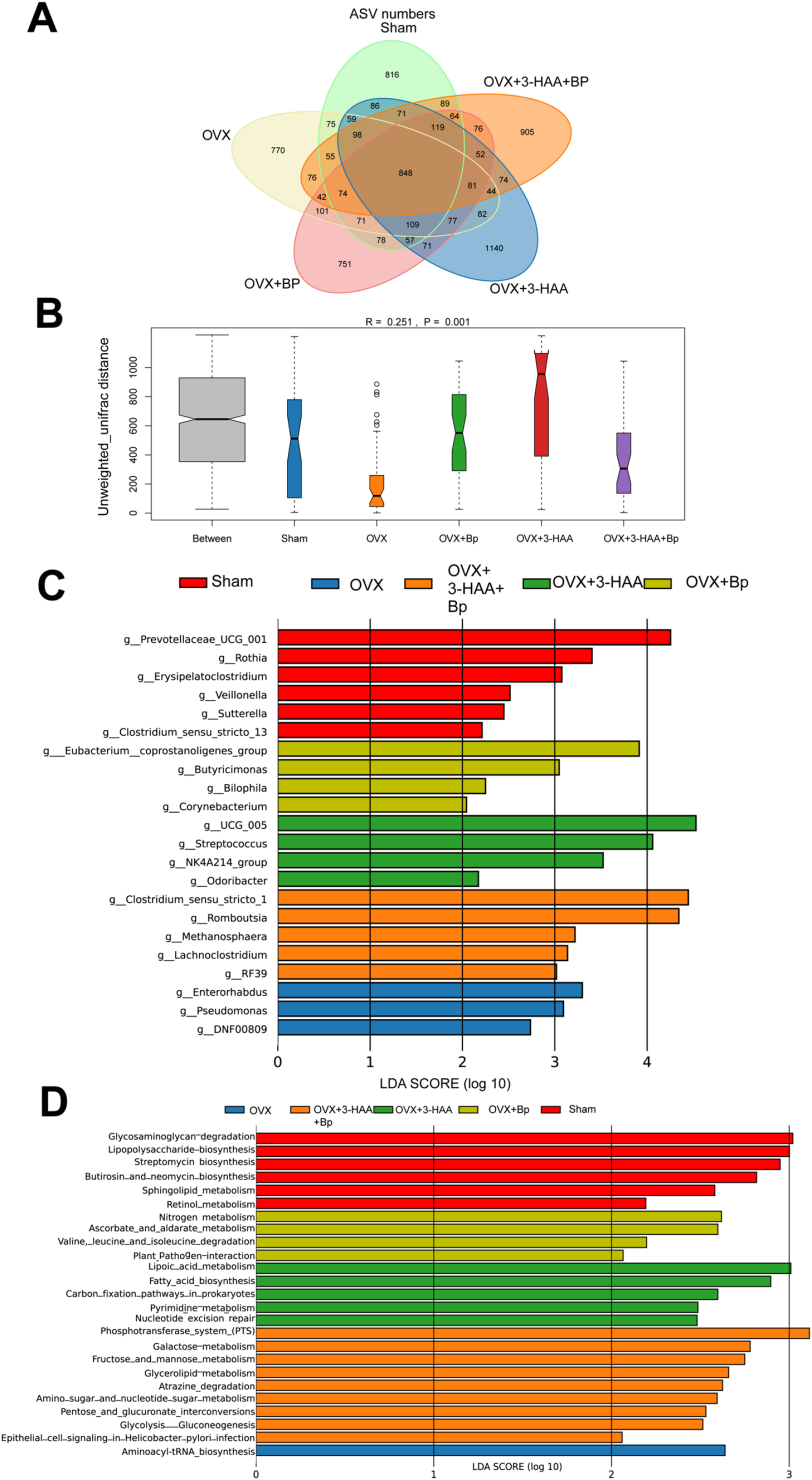
The combined use of *Bp* and 3-HAA effectively improves the gut microbiota structure and functionality in OVX rats. **A** Venn diagram of the number of ASVs. **B** Microbial β-diversity analysis among different groups based on unweighted uniFrac distance. **C** Detection of significantly differentially abundant taxa among different groups through Lefse analysis. **D** KEGG functional prediction analysis of gut microbiota among different groups

To further analyze whether the combined use of *Bp* and 3-HAA can improve the structure and functionality of gut microbiota in OVX rats, we conducted microbiota analysis of fecal samples from different groups of rats using 16S rRNA sequencing technology. Based on ASV data, we analyzed the community composition of the samples. Compared to the Sham group, the fecal microbiota diversity was significantly reduced in the OVX group. However, compared to the OVX group, the administration of *Bp*, 3-HAA, and the combination of 3-HAA and *Bp* resulted in an increase in the diversity of gut microbiota in OVX rats. Compared to OVX rats treated with *Bp* alone, the group receiving the combination therapy exhibited an increased diversity of fecal microbiota. However, the group receiving 3-HAA alone had a greater variety of bacterial species in the fecal microbiota compared to the combination therapy group (Fig. 4A). Based on the analysis of unweighted_unifrac distance and Anosim, the *R*-value of β-diversity was calculated as 0.251, which falls between 0 and 1, and the *P* value was found to be 0.001, less than 0.05 (Fig. 4B). These results indicate that the differences between the groups are greater than the differences within the groups, and the grouping is statistically meaningful.

To explore the differences in microbial composition between the groups at the genus level, we performed a linear discriminant analysis (LDA) effect size (Lefse) analysis. Lefse analysis allows us to estimate the impact of each species’ relative abundance on the observed differences. A significance threshold of LDA score > 2 and a *P* value <0.05 were applied as selection criteria. At the genus level, the analysis revealed that *Prevotellaceae_ucg_001*, *Rothia*, *Erysipelatoclostridium*, *Veillonella*, *Sutterella* and *Clostridium_sensu_stricto_13* were significantly enriched in the Sham group. OVX rats exhibited significant enrichment of *Enterorhabdus*, *Pseudomonas*, and *DNF00809*, suggesting their potential use as fecal microbial markers in PMO rats. In the OVX+*Bp* group, *Eubacterium_coprostanoligenes_group*, *Butyricimonas*, *Bilophila* and *Corynebacterium* were enriched. In the OVX+3-HAA group, *UCG_005*, *Streptococcus*, *NK4A214_group* and *Odoribacter* were among the enriched microbial genera. In the OVX+3-HAA+*Bp* group, the enrichment of *Clostridium_sensu_stricto_1*, *Romboutsia*, *Methanophaera*, *Lachnoclostridium* and *RF39* in the gut microbiota was observed (Fig. 4C).

To further elucidate the functional changes in the gut microbiota caused by alterations in the gut microbiota structure among different groups, we conducted functional prediction analysis using the KEGG database. We screened significant differences in KEGG pathways among the groups based on the LDA score > 2 criteria and *P* value <0.05. The results of these categories indicate that the differences in gut microbiota functionality associated with changes in gut microbiota structure among the groups mainly involve genetic, immune, and metabolic pathways related to the metabolism of carbohydrates, lipids, and amino acids. The application of *Bp* and 3-HAA is primarily associated with the enrichment of immune and nucleotide pathways, as well as metabolic pathways related to carbohydrates, lipids, and amino acids. We observed a significant enrichment of aminoacyl-tRNA biosynthesis in the OVX group, which may be associated with the progression of PMO (Fig. 4D). Based on the above results, it can be concluded that the combined use of *Bp* and 3-HAA improves the structure and functionality of the gut microbiota in OVX rats.

### The combined use of *Bp* and 3-HAA prevented OVX by regulating the microbiota-Th17/Treg immune axis

**Fig. 5:**
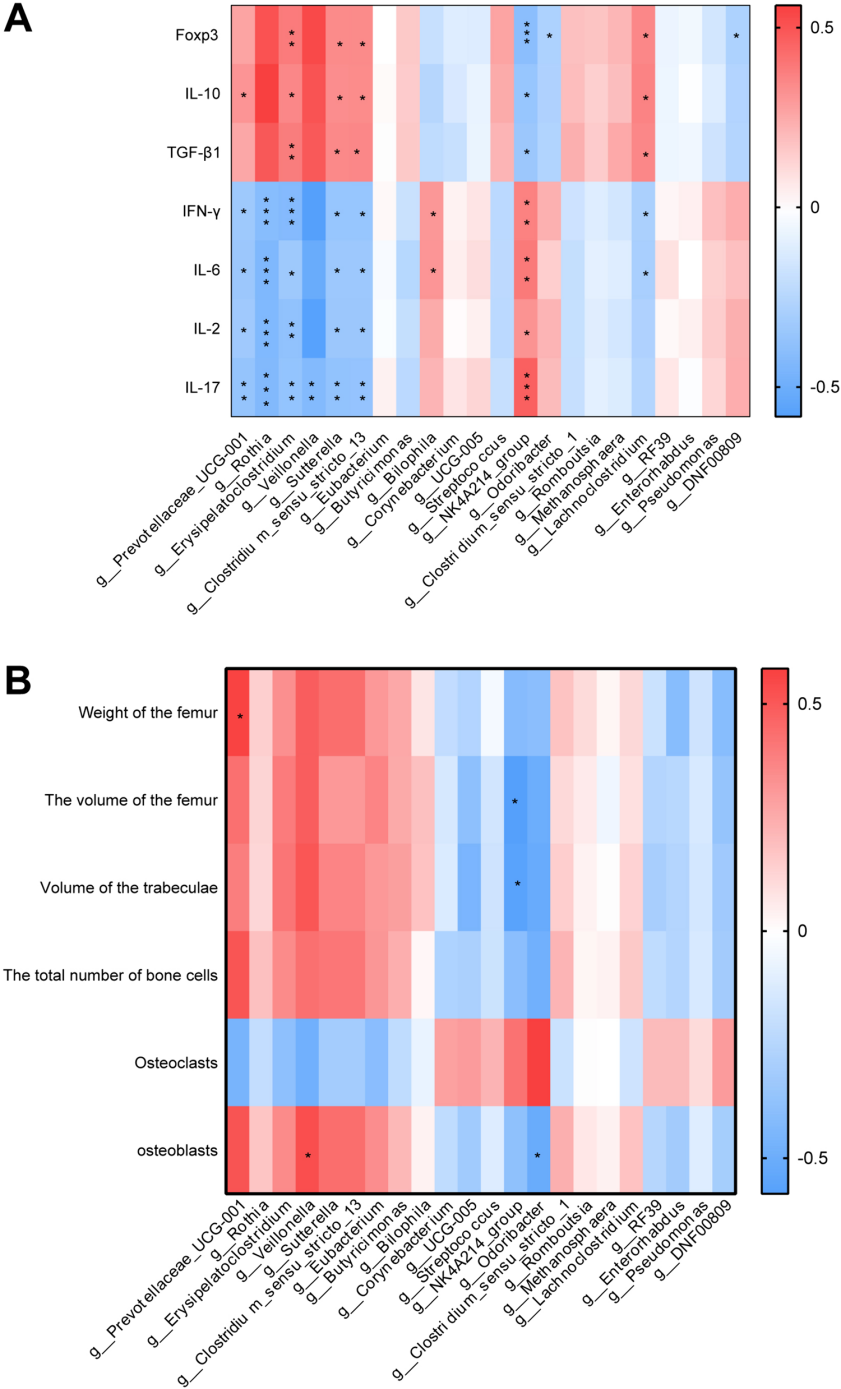
The combined use of *Bp* and 3-HAA prevents OVX by modulating the gut microbiota-Th17/Treg immune axis. **A** The correlation between differentially abundant microbes and Th17/Treg immune markers was evaluated using Spearman analysis. **B** A heatmap was generated to depict the Spearman correlation between differentially abundant microbes and femoral stereological parameters indicators across the different groups. * *P* < 0.05, ** *P* < 0.01, *** *P* < 0.005. Non-significant comparisons are indicated by no asterisk

To explore whether the combined administration of *Bp* and 3-HAA prevents PMO by modulating the gut microbiota-Th17/Treg immune axis, we examined the correlations between differentially abundant microbes at the genus level, Th17/Treg immune factors, and the stereological parameters of the femur in OVX rats across the experimental groups. A Spearman correlation analysis was performed to filter out the significant correlations between the selected microbial taxa (Fig. 4C) and the Th17/Treg cell cytokines and bone stereological parameters. Microbes were selected based on a significance level of *P* < 0.05. Heatmaps were generated to visualize the results. A darker red color in the heatmap indicated a stronger positive correlation between the two variables. A darker blue color in the heatmap indicated a stronger negative correlation between the two variables. At the genus level, the bacterial taxa *Prevotellaceae_UCG-001* was markedly positively correlated with IL-10 and negatively correlated with IFN-γ, IL-6, IL-2, and IL-17. The bacterial taxa *Rothia* was significantly negatively correlated with IFN-γ, IL-6, IL-2, and IL-17. The bacterial taxa *Erysipelatoclostridium*, *Sutterella*, and *Clostridium_sensu_stricto_13* were positively correlated with Foxp3, IL-10, and TGF-β1, and negatively correlated with IFN-γ, IL-6, IL-2, and IL-17. The bacterial taxa *Veillonella* was negatively correlated with IL-17. The bacterial taxa *Bilophila* was positively correlated with IFN-γ and IL-6. The *NK4A214_group* showed a significant negative correlation with Foxp3, IL-10 and TGF-β1, while positively correlated with IFN-γ, IL-6, IL-2, and IL-17. The *Odoribacter* was negatively correlation with Foxp3. The *Lachnoclostridium* was positively correlated with Foxp3, IL-10, and TGF-β1 and negatively correlated with IFN-γ and IL-6. The *DNF00809* was negatively correlated with Foxp3 (Fig. 5A). The *Prevotellaceae_UCG-001* was positively correlated with femoral weight. The *Veillonella* was positively correlated with osteoblast cell count. The *NK4A214_group* was negatively correlated with femoral volume and trabecular bone volume. The *Odoribacter* was positively correlated with osteoclast cell count (Fig. 5B). Based on the previous findings, the combined administration of *Bp* and 3-HAA prevents OVX by modulating the microbiota-Th17/Treg immune axis.

## Discussion

PMO is a chronic disease that causes the weakening of bones, significantly affecting the quality of life in middle-aged and older women [38]. Quercetin could potentially improve bone loss in PMO rats, possibly due to the enrichment of *Bp* [39, 40]. Clinical studies confirmed that 3-HAA was downregulated in patients with osteoporosis [41]. This study demonstrated that the combined use of 3-HAA and *Bp* prevented PMO in OVX rats by modulating the gut microbiota-Th17/Treg immune axis.

SCFAs are metabolites derived from the gut microbiota and are widely recognized for playing a crucial role in improving human health [42]. *Bp* is a safe butyrate-producing microorganism [20]. Our previous research found that *Bp* was enriched in PMO rats undergoing quercetin treatment. Studies indicated that a decrease in 3-HAA concentration was found to be one of the contributing factors to PMO [41]. We confirmed at the level of bone tissue three-dimensional parameters and osteoblast/osteoclast levels that supplementation of both *Bp* and 3-HAA reduced bone loss in OVX rats, with better effects observed with combined administration. Autophagy plays a significant role in regulating bone cell fate and has been shown to have a certain impact on bone metabolism regulation [43]. Bone cell homeostasis, including the maintenance of cellular function, differentiation, and stress response, was tightly regulated by autophagy [44]. Our results showed that the combined administration of *Bp* and 3-HAA promoted the restoration of the disrupted microstructure of the femoral tissue and inhibited autophagy in the femoral tissue. The gut microbiota, a crucial ecological system within the human body, was closely associated with PMO [45]. A recent study found that genes encoding estrogen receptors, xenobiotic processing genes, as well as genes related to gut homeostasis and bile acid biosynthesis in the gut and liver were regulated by estrogen. Estrogen deprivation led to dysregulation of gut homeostasis and bile acid enterohepatic circulation [46]. We hypothesized that the pathological progression of PMO could also be closely associated with dysregulation of gut homeostasis and bile acid enterohepatic circulation. A study confirmed that supplementation with beneficial bacterial strains could modulate the gut microbiota and may effectively prevent bone loss [47]. He et al. found that patients with PMO had a decreased abundance and diversity of gut microbiota in their fecal samples [48]. In our study, we also observed a decrease in the abundance and diversity of gut microbiota in the fecal samples of OVX rats. After treatment with *Bp* and 3-HAA, the diversity of gut microbiota structure increased in OVX rats. Based on the LDA score, *Enterorhabdus* and *Pseudomonas* bacteria may serve as potential microbial markers for the progression of PMO in OVX rats. The enrichment of *Prevotellaceae_UCG_001*, *Rothia*, and *Erysipelatoclostridium* gut microbiota of the Sham group is likely beneficial in preventing PMO occurrence. Enrichment of bacterial species such as *Clostridium_sensu_stricto_1*, *Romboutsia*, *Methanosphaera* and *Lachnoclostridium* may potentially contribute to the therapeutic effects of *Bp* and 3-HAA in treating PMO. According to KEGG analysis, *Bp* and 3-HAA improved the functional profile of gut microbiota in OVX rats, particularly in metabolism, genetic pathways, and immune pathways.

In this study, we noted that gut microbial taxa, such as *Prevotellaceae_UCG-001* and *Veillonella*, were positively correlated with parameters related to bone formation in the femurs of OVX rats. In contrast, a positive correlation was found between these microbial taxa, such as *Odoribacter* and the number of osteoclasts in the femoral tissue. The *NK4A214_group* was negatively correlated with osteogenesis in OVX rats. This suggested that the enrichment of harmful gut microbial groups can promote the development of PMO, while probiotics can ameliorate PMO progression. *Bp* contributed to the enrichment of gut microbiota in OVX rats. The isolated use of 3-HAA may lead to the proliferation of harmful bacterial populations. Still, when combined with *Bp*, it could promote the colonization of more beneficial bacteria in the gut. Based on KEGG analysis, *Bp* and 3-HAA contributed to maintaining glucose and lipid metabolism as well as amino acid metabolism in PMO. These metabolic pathways are closely related to the enterohepatic circulation of bile acids. Therefore, our study demonstrated that combined treatment with *Bp* and 3-HAA could alleviate the disruption of gut microbiota in PMO caused by estrogen deprivation.

It has been established in the scientific community that estrogen has regulatory effects on the immune system. Recently, studies indicated that T lymphocytes served as mediators in the effects of estrogen on bone mass [49]. The imbalance of Th17/Treg promoted the progression of PMO [11]. Yu et al. [50] demonstrated that OVX promoted the migration of Th17 cells from the gut to the bone marrow, while the presence of certain gut microbial taxa inhibited the accumulation of Th17 cells in the bone marrow. Using probiotics to manipulate the gut microbiota can reduce inflammation and treat PMO with minimal side effects [21, 51]. Rhubarb Peony Decoction functioned in ulcerative colitis by regulating the gut microbiota and restoring the balance between Th17 and Treg cells. The increased abundance of *Bp* was one of the major beneficial microbial strains involved in this process [22]. Increasing evidence suggests that the redox-active compound 3-HAA may benefit cellular function in the immune system [52]. In the past, it was found that 3-HAA could reduce the number of Th17 cells [26]. Our research findings aligned with the content as we found that treatment with *Bp* and 3-HAA, either alone or in combination, could reduce the population of Th17 cells in the peripheral blood and spleen tissue of OVX rats and increase the number of Treg cells. At the same time, *Bp* and 3-HAA suppressed the expression of IL-17 in the femurs of OVX rats and increased the expression of Foxp3. These findings indicate that the combined treatment with *Bp* and 3-HAA can restore the disrupted Th17/Treg balance in OVX rats. Additionally, the enrichment of microbial taxa such as *Sutterella*, *Clostridium_sensu_stricto_13* and *Lachnoclostridium* was negatively correlated with Th17-related inflammatory cytokines and positively correlated with Treg cell immune factors. Based on the Lefse analysis results of our study, the *Lachnoclostridium* group was significantly enriched in the *Bp* and 3-HAA combination treatment group. The enrichment of microbial communities induced by BP and 3-HAA can improve the Th17/Treg imbalance in PMO by suppressing Th17-related pro-inflammatory factors such as IL-17, IL-6, IFN-γ, and IL-2 production, and promoting the release of Treg-related anti-inflammatory factors including IL-10, TGF-β, and Foxp3.

In vivo, we observed that the combined treatment of *Bp* and 3-HAA yielded better therapeutic effects in PMO. However, the 16S analysis results revealed that the gut microbiota structure of the combined treatment group in OVX rats was less diverse compared to the single treatment groups. Indeed, these results may be influenced by the interactions between the drugs and different microbial taxa. The presence of both *Bp* and 3-HAA may have altered the composition and abundance of certain bacterial species, leading to a less diverse gut microbiota structure in the combined treatment group. Further clinical observations are needed to evaluate the effectiveness of different administration methods. This study’s correlation analysis reveals that the enriched microbiota in the Sham group (such as *Prevotellaceae_UCG_001*, *Rothia*, and *Erisipelatoclostridium*) and the enriched microbiota in the group treated with Bp and 3-HAA (including *Clostridium_sensu_stricto_1*, *Romboutsia*, and *Lachnoclostridium*) are positively correlated with osteogenesis and anti-inflammatory factors associated with Treg cells, and negatively correlated with bone resorption and pro-inflammatory immune factors related to Th17, although the correlations are relatively weak. In the upcoming research, we plan to select several microbiotas that show significant correlations and establish a PMO-related risk model through bioinformatics analysis, aiming to further investigate beneficial bacteria that can improve the condition of PMO. This will help identify more potential targets for probiotic adjunctive therapy in PMO.

## Conclusion

In conclusion, our study elucidates the role of *Bp* and 3-HAA in treating PMO induced by estrogen deficiency. The combination therapy of *Bp* and 3-HAA can prevent PMO in OVX rats by regulating the gut microbiota and Th17/Treg immune balance. We hope this study can provide valuable insights into applying probiotic combination therapy for PMO.

## Data Availability

The data used to support the findings of this study are included within the article.
